# Psychometric validity of the Shirom-Melamed Burnout Measure and the Burnout Assessment Tool: a systematic review

**DOI:** 10.2478/aiht-2023-74-3769

**Published:** 2023-12-29

**Authors:** Yara Shoman, Roy Hostettler, Irina Guseva Canu

**Affiliations:** University of Lausanne Centre of Primary Care and Public Health (Unisanté), Lausanne, Switzerland

**Keywords:** BAT, occupational burnout, psychometric properties, patient-reported outcome measures, SMBM, BAT, mjere ishoda koje opisuje pacijent, profesionalno izgaranje, psihometrijska svojstva, SMBM

## Abstract

In the absence of internationally recognised standardised criteria, several patient-reported outcome measures (PROMs) have been developed to measure occupational burnout. The aim of this study was to extend our 2021 review of the psychometric validity of five PROMs to the Shirom-Melamed Burnout Measure (SMBM) and the Burnout Assessment Tool (BAT). To do that we ran a systematic literature search in the MEDLINE, PsycINFO, and Embase databases following our previous methodological framework and the COnsensus-based Standards for the selection of health Measurement Instruments (COSMIN). We assessed the level of evidence using the Grading of Recommendations, Assessment, Development, and Evaluation (GRADE) guideline. We identified 694 publications on SMBM and 421 on BAT, but the final review includes eight papers on SMBM and three on BAT. Of the seven psychometric properties assessed for SMBM, content, structural, and criterion validity were rated as insufficient, whereas the quality of evidence for construct and internal consistency was high and moderate, respectively. Of the nine psychometric properties assessed for BAT, content, structural, criterion, and construct validity was moderate and internal consistency was high. One limitation of this study is that we did not assess cross-cultural validity, because the number of studies reviewed is too small and content validity can only be assessed based on the original PROM version rather than translation. To conclude, BAT is superior to SMBM in terms of psychometric validity, but the quality of evidence for some properties is low or very low, suggesting a need for additional validation studies.

## Abstract (sl)

U nedostatku međunarodno priznatih standardiziranih kriterija osmišljeno je nekoliko mjera ishoda koje opisuje pacijent (engl. *patient-reported outcome measures*, krat. PROM) za mjerenje profesionalnog izgaranja. Cilj je ovog ispitivanja bio proširiti naš prethodni pregled psihometrijske valjanosti pet PROM-ova iz 2021. na upitnike *Shirom-Melamed Burnout Measure* (SMBM) i *Burnout Assessment Tool* (BAT). U tu smo svrhu sustavno pretražili literaturu dostupnu u bazama podataka *MEDLINE*, *PsycINFO* i *Embase,* služeći se metodološkim okvirom iz prethodnog ispitivanja te tzv. COSMIN standardima za odabir mjernih instrumenata temeljenima na konsenzusu (izv. *COnsensus-based Standards for the selection of health Measurement Instruments*). Razinu dokaza ocjenjivali smo pomoću smjernica *Grading of Recommendations, Assessment, Development, and Evaluation* (GRADE). Pretragom su izdvojene 694 publikacije za SMBM te 421 za BAT, no ovaj konačni pregled obuhvaća osam članaka o SMBM-u i tri o BAT-u. Od sedam ocijenjenih psihometrijskih svojstava za SMBM, valjanost sadržaja i strukture kriterija ocijenjeni su nedovoljnom ocjenom, a kakvoća dokaza za konstrukt odnosno interna konzistencija ocijenjeni su kao snažni odnosno umjereni. Od devet psihometrijskih svojstava za BAT, valjanost sadržaja te strukture kriterija i konstrukta ocijenjeni su umjerenima, a interna konzistencija snažnom. Jedno od ograničenja ovoga ispitivanja svakako je činjenica da nismo ocjenjivali međukulturnu valjanost (engl. *cross-cultural validity*) budući da je količina pregledanih ispitivanja premalena, a valjanost sadržaja može se ocijeniti isključivo na temelju izvorne inačica PROM-a, a ne njezina prijevoda. Zaključno, BAT ima bolju psihometrijsku valjanost od SMBM-a, no kakvoća je dokaza za pojedina svojstva niska ili veoma niska, što upućuje na daljnja istraživanja valjanosti.

In May 2019, the WHO recognised burnout as an occupational phenomenon resulting from a “chronic stress at the workplace that has not been successfully managed” (1). The 11^th^ revision of the International Classification of Diseases (ICD-11) classifies burnout among “factors influencing health status or contact with health services” (2). The latter includes reasons for using health services other than disease. In its advanced stage, burnout shares several symptoms with depression but is mostly seen as a risk factor for depression or a mediator between job stress and depression (3). While depression has been established as a leading cause of disability worldwide and a major contributor to the overall global burden of disease for a while (4, 5), burnout is recognised as a disease only by a few countries (6). Affecting all occupational sectors on all continents, burnout is particularly prevalent in the health care sector (7, 8, 9, 10, 11, 12, 13, 14). In the USA, physician burnout was declared “public health crisis” in 2019 (15).

Since the breakout of COVID-19 pandemic, physicians have been reporting burnout in all medical specialties at unprecedented rates (16). However, it has also been affecting mental health of other front-line or essential workers (17, 18, 19, 20, 21) and all workers in general. By aggravating mental health determinants in the working and living environment, the COVID-19 crisis triggered a 27.6 % increase in the incidence of major depressive disorder globally (22). The pandemic's impact on burnout rates, however, presents a challenge, as, unlike the depressive disorder and despite recent efforts (6, 23), there are still no standardised and internationally accepted criteria to assess occupational burnout. Instead, occupational burnout is mostly measured using patient-reported outcome measures (PROMs) (24).

There are about a dozen PROMs for occupational burnout, which raises the issue of inconsistencies in reporting and difficulties in comparing and combining the findings for systematic reviews and meta-analyses (25). Moreover, the psychometric quality of PROMs varies considerably (25, 26), which is why we systematically reviewed five PROMs considered valid for measuring occupational burnout in mental health professionals (27) to identify the best ones. That review (28) included the Maslach Burnout Inventory (MBI), the Pines’ Burnout Measure, the Psychologist Burnout Inventory, the Oldenburg Burnout Inventory (OLBI), and the Copenhagen Burnout Inventory (CBI) and concluded that only CBI and, to a lesser extent, OLBI had sufficient content validity, which is the most important psychometric property of a PROM (29).

This study extends our review to two more PROMs, the Shirom-Melamed Burnout Measure (SMBM) and Burnout Assessment Tool (BAT) using the same methodology. The difference is that these two PROMs have been developed for all occupations and integrate all dimensional sub-scores into a total score. This gives them an important advantage over the other PROMs, as it allows determining a cut-off value to identify occupational burnout cases.

## MATERIALS AND METHODS

This systematic review followed the Preferred Reporting Items for Systematic Reviews and Meta-Analyses (PRISMA) checklist (30) and the updated review protocol available in the international PROSPERO database (31).

### Eligibility criteria

For each PROM we conducted a separate literature search and review. The analysis included research articles reporting quantitative testing of psychometric properties of the original full PROM version (not the shortened ones) with a sample size of >100 participants. We excluded studies for which no full text could be found, studies where a PROM was compared to another PROM, one of the two burnout PROMs was compared to a PROM not included in this review, and whose sample were non-professional participants (e.g., students).

### Data sources and search terms

Before starting the literature search, we consulted the COnsensus-based Standards for the selection of health Measurement Instruments (COSMIN) database of systematic reviews of outcome measurement instruments (32) to identify available studies using SMBM or BAT but found none. We then continued search of the following databases: MEDLINE, PsycINFO, and Embase.

For SMBM, we searched studies published between January 2000 and January 2023. As BAT was introduced in 2020, literature search included all studies since 2020. For both PROMs it consisted of free-text words specifying terms focusing on the PROM of interest (e.g., BAT), terms related to the validation of the PROM, and a combination of the two first search strings results. One additional search string served to remove duplicates.

In addition, we checked reference lists in articles and reviews retrieved in our electronic search for any additional studies to include in this review, and contacted PROM authors to check the completeness of our search if needed.

### Study selection

Study selection followed a three-step process done by two independent reviewers. First, the reviewers eliminated remaining duplicates. Second, they examined the title and abstract of each article using the Rayyan application (33) and kept or rejected them based on the above-mentioned inclusion and exclusion criteria. Unclassifiable articles, about half a dozen, were also retained for full-text reading. Third, the reviewers read all included full text articles. For each of the three steps, reviewers discussed all discrepancies in the assessment of the studies and, when needed, consulted a third reviewer.

### Data extraction and management

We extracted the data using the previously developed standardised form (28) for extraction of the following data: study identification, sample characteristics, and statistical methods used to assess psychometric properties, quantitative results for each property, and authors’ interpretations. All data were extracted by one reviewer and double-checked by the other. All disagreements were discussed in the presence of the third reviewer.

### Validity assessment and quality grading

We replicated the procedure described in detail in our previous article (28). First, we counted the psychometric properties assessed by included articles for each of the two PROMs. Second, we assessed PROM's content validity following a COSMIN user manual (32). Third, we examined the reported quantitative results of the remaining psychometric properties and interpreted them using the protocol described by Marca et al. (34). We then compared the authors’ result interpretation with ours to see to which extent these two interpretations agree. Complete agreement means no discrepancy between them. Partial agreement entails differences in cut-off values, e.g., when a correlation of 0.50 is considered strong by the authors but moderate by our reviewers. Disagreement arises when the authors interpret a model as acceptable and our reviewers as not acceptable, based on goodness-of-fit indices. Agreement assessment was not possible when the authors’ interpretation was missing. Fourth, we assessed the risk of bias of each validation study following the method described by Mokkink et al. (35). Finally, we graded the level of validity for each psychometric measure as very low, low, moderate, or high, depending on the risk of bias, consistency, directness, and precision of results obtained by each PROM (32).

## RESULTS

### Construct definition and PROM description

SMBM defines burnout as “an affective state characterised by one's feelings of being depleted of one's physical, emotional and cognitive energies” (36). This construct is based on the conservation of resources theory, according to which people strive to obtain, retain, and protect their resources, which can be material, social, and energy (37). Energy resources include physical, emotional, and cognitive energy. Burnout is thus a combination of physical fatigue, emotional exhaustion, and cognitive weariness, the three dimensions of SMBM. The latest standard version of SMBM comprises 14 items: six on physical fatigue, three on emotional exhaustion, and five on cognitive weariness. All items are scored on a 7-point frequency scale ranging from 1 (almost never) to 7 (almost always). The mean score across the 14 items is used as a total score of burnout.

BAT, on the other hand, views burnout as “a work-related state of exhaustion that occurs among employees, which is characterised by extreme tiredness, reduced ability to regulate cognitive and emotional processes, and mental distancing. These four core dimensions of burnout are accompanied by depressed mood as well as by non-specific psychological and psychosomatic complaints” (38). In its original version BAT consists of a core part (BAT-C), which includes 23 items covering four core dimensions [exhaustion (eight items), mental distance, cognitive impairment, and emotional impairment (five items each)] and the secondary part (BAT-S) with three symptom dimensions (depressed mood, psychological distress, and psychosomatic complaints) each including five items (39). All items are scored on a 5-point frequency scale ranging from 1 (never) to 5 (always).

**Figure 1:**
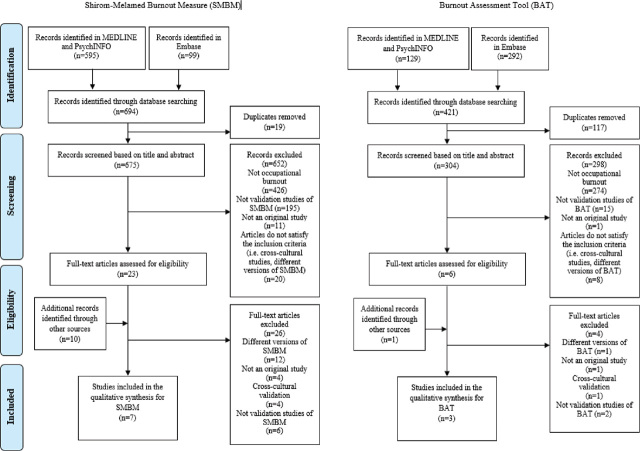
Selection of studies for review

Figure 1 summarises the study selection process. For SMBM, we selected eight articles (3, 36, 40, 41, 42, 43, 44, 45) and for BAT two article and one report (38, 39, 46).

### PROM validation completeness and psychometric validity

For SMBM we identified six psychometric properties (out of eleven) that had been assessed by analysed papers (Table 1). While Shirom described the construct (36) with reference to his earlier work on the construct origin (47), none describes SMBM development and validation or specify the target population. Instead, they claim that SMBM can be used for all occupations and purposes (i.e., discriminative, evaluative purpose, and/or predictive). This is why we considered the PROM's design and development inadequate. Its structural validity is also inadequate, since the papers consider only two of the three dimensions: physical fatigue and cognitive weariness (43). Internal consistency or homogeneity can be considered adequate (Table 1), but four of the seven papers (3, 40, 41, 42, 43, 44, 45) do not report internal consistency statistics for each dimension separately, as required by COSMIN. Four studies (40, 41, 42, 45) report the PROM's reliability, but their assessments are dubious in terms of the time interval between administrations, which is too long and, more importantly, in terms of the poor choice of the statistical method. The same concern goes for the measurement error, as no paper but one (41) report standard error of measurement, smallest detectable change, or limits of agreement. Construct validity assessment was adequate, demonstrating convergent validity with burnout measured by MBI-GS (43) and divergent and discriminate validity with depressive symptoms (3, 45). An important validation effort was dedicated to the SMBM predictive validity using inflammation biomarkers (44), type 2 diabetes (42), musculoskeletal pain (40), and depressive symptoms (3, 45) as health outcomes.

**Table 1:** PROM description, initial validation, and validity of statistical analysis

	**Shirom-Melamed Burnout Measure**	**Burnout Assessment Tool**
Authors	Shirom and Melamed	Schaufeli, Desart, and De Witte
Aim	Screening for employees at risk of burnout	Screening for employees at risk of burnout
Year of publication	2003	2020
Country	Israel	Belgium
Language of original version	Hebrew and English	Flemish Dutch and English
Population	Working population	Working population
Dimensions (Number of items)	Physical fatigue (6)	Exhaustion (8)
	Cognitive weariness (5)	Mental distance (5)
	Emotional exhaustion (3)	Cognitive impairment (5)
		Emotional impairment (5)
Number of analysed articles	7	3
Number of psychometric prosperities assessed by these articles	6/11	9/11
Face validity	-	1
Content validity	-	1
Predictive validity	5	-
Concurrent validity	-	-
Convergent validity	1	1
Discriminant validity	2	1
Exploratory factorial validity	-	2
Confirmatory factorial validity	1	2
Stability	4	1
Homogeneity	7	3
Sensitivity	-	1
	**Comparison between authors' and reviewers' interpretation**	
Complete agreement	6/7	1/3
Partial agreement	1/7	2/3
Disagreement	0/7	0/3

For BAT we identified nine of the eleven psychometric properties assessed by the analysed papers (Table 1). BAT development, construct, its origin and conceptual framework, target population, and the purpose are all very well described. Yet, in contrast to SMBM, the theory underpinning the BAT construct appears less formalised but more as a response to the drawbacks of preceding burnout PROMs (48). People at work are the target population of BAT. However, the face validity assessment of the first and revised versions have been conducted in a sample of general practitioners, occupational physicians, and psychologists (n=49) and among the PROM authors (n=3), and we found no evidence that the cognitive interview study or other pilot tests involved other workers. Although the studied professionals can be at risk of burnout, it seems doubtful that they are representing the target population for which the PROM was developed. Concept elicitation and relevance, comprehensiveness, and comprehensibility are insufficiently described to determine whether group meetings or interviews were recorded and transcribed verbatim or at least part of the data were coded independently, or appropriate qualitative method was used to assess the comprehensibility of PROM instructions, items, response options, and recall period, or whether at least two researchers were involved in the analysis. Therefore, we qualified relevance, comprehensiveness, and comprehensibility as doubtful, although most of the PROM development criteria were judged as very good or adequate. The structural validity of BAT was assessed using confirmatory factor analysis, and we partly disagreed with the authors’ interpretation of results (data available on request). One paper (46) presents Rasch analysis and strengthens the evidence of BAT's structural validity, but only for the core part. Internal consistency has been evaluated as high by all studies (data available on request). One report (39) presents BAT's inter-rater and a test-retest reliability and a one-year stability coefficient for the latter. The appropriateness of this time interval seems adequate, but considering the COSMIN-recommended two-week interval, it needs a justification. Furthermore, the statistics used (Pearson's correlation coefficients and Cohen's d) is inadequate by COSMIN standards. Even so, the construct and criterion validity were assessed in an appropriate and comprehensive manner (data available on request). Convergent validity of BAT-C was assessed and confirmed by comparison with MBI-GS and OLBI (Table 2). Discriminant validity was examined with respect to the lack of work engagement, work addiction, and job boredom for both BAT-C and BAT-S, and we slightly disagreed with the authors’ interpretation of results (data available on request).

**Table 2:** Comparison of the two burnout PROMs against the COSMIN guidelines

	**Shirom-Melamed Burnout Measure**		**Burnout Assessment Tool**	
	**Overall rating**	**Quality of evidence**	**Overall rating**	**Quality of evidence**
Content validity	−	Very low	+	Moderate
Relevance	−	Very low	+	Moderate
Comprehensiveness	−	Very low	+	Moderate
Comprehensibility	−	Very low	+	Moderate/Low
Structural validity	−	Very low	+	Moderate
Internal consistency	+	Moderate	+	High
Reliability	+	Low	+	Low
Measurement error	?	Very low	−	Low
Criterion validity	−	Very low	+	Moderate
Construct validity	+	High	+	Moderate
Responsiveness	?	Very low	?	Very low

+ sufficient psychometric property; − insufficient psychometric property; ? undetermined

### Risk of bias and overall evidence in view of the COSMIN guidelines

Content validity for SMBM was not assessed by any of the analysed papers, which is why we find SMBM content validity insufficient and the evidence level very low (Table 2). Our reviewers initially graded the relevance, comprehensiveness, and comprehensibility of BAT as sufficient, but given the potential indirectness of the study population, we downgraded the evidence level of comprehensibility to moderate/low. As the only study assessing structural validity of the SMBM (43) had a high risk of bias, the level of evidence for this psychometric property of the SMBM is very low. BAT, in contrast, shows sufficient structural validity only for BAT-C, and the quality of evidence is moderate. Internal consistency was sufficient in both PROMs, though the level of evidence was better for BAT. Both PROMs also showed a sufficient reliability, but the level of evidence was low (data available on request). Construct validity was sufficient, with a moderate level of evidence for BAT, because of limited number of convergent/discriminant validity studies and partially inconsistent results. As for SMBM, several high-quality studies (3, 40, 42, 45) confirm the tested hypothesis, which is why we consider the level of evidence high (Table 2). Finally, we could not assess responsiveness, as no analysed studies reported relevant statistics to do so.

## DISCUSSION

Our assessment of the PROM validity evidence suggests that BAT is more valid than SMBM. Furthermore, its validity surpasses that of the five PROMs assessed in our earlier study (28), especially in view of its content and criterion validity. The latter is actually unique for BAT.

Although several recent studies report cut-off values and further validity assessments of SMBM, they were conducted on later SMBM versions, translated into Swedish and German and extensively revised (49, 50, 51). Therefore, we could not include these studies in this review.

As BAT is the most recent PROM for occupational burnout, its development and validation steps have been better reported, despite low-level of quality evidence for some psychometric properties. Newer PROMs have obviously been developed to tackle the drawbacks of their predecessors and to perform better. SMBM was developed long before methodological guidelines for PROM validation became available, which is why it may have failed in some psychometric respects. However, the resource conservation theory that underpins SMBM is still valid and widely applied in psychology (52, 53), and the PROM is highly predictive and discriminates remarkably well against depressive symptoms (3), which cannot be said for BAT yet, as no assessment of the kind has been reported. Furthermore, the structural validity of BAT-S leaves much to be desired for BAT to discriminate burnout well from other affective states, such as depression or anxiety.

Our systematic review has certain limitations, the first being that it evaluated content validity of the original PROM versions only. Secondly, the number of validity assessment studies is small, for BAT in particular. More will be known as more methodologically robust validation studies are reported in the future.

## CONCLUSION

Regardless of the above limitations, our findings single out BAT as the most complete burnout PROM with sufficient content, structural, construct, and criterion validity and internal consistency. However, the quality of evidence for some of these properties is low or very low, suggesting a need for additional validation studies.
